# Arsenic, Organic Foods, and Brown Rice Syrup

**DOI:** 10.1289/ehp.1104619

**Published:** 2012-02-16

**Authors:** Brian P. Jackson, Vivien F. Taylor, Margaret R. Karagas, Tracy Punshon, Kathryn L. Cottingham

**Affiliations:** 1 Trace Element Analysis Core Laboratory, Department of Earth Sciences, Dartmouth College, Hanover, New Hampshire, USA; 2 Department of Community and Family Medicine, Section of Biostatistics and Epidemiology, Dartmouth Medical School, Lebanon, New Hampshire, USA; 3 Department of Biological Sciences, Dartmouth College, Hanover, New Hampshire, USA

**Keywords:** arsenic, baby formula, brown rice syrup, cereal bars, energy bars, organic foods, speciation

## Abstract

Background: Rice can be a major source of inorganic arsenic (As_i_) for many sub-populations. Rice products are also used as ingredients in prepared foods, some of which may not be obviously rice based. Organic brown rice syrup (OBRS) is used as a sweetener in organic food products as an alternative to high-fructose corn syrup. We hypothesized that OBRS introduces As into these products.

Objective: We determined the concentration and speciation of As in commercially available brown rice syrups and in products containing OBRS, including toddler formula, cereal/energy bars, and high-energy foods used by endurance athletes.

Methods: We used inductively coupled plasma mass spectrometry (ICP-MS) and ion chromatography coupled to ICP-MS to determine total As (As_total_) concentrations and As speciation in products purchased via the Internet or in stores in the Hanover, New Hampshire, area.

Discussion: We found that OBRS can contain high concentrations of As_i_ and dimethyl-arsenate (DMA). An “organic” toddler milk formula containing OBRS as the primary ingredient had As_total_ concentrations up to six times the U.S. Environmental Protection Agency safe drinking water limit. Cereal bars and high-energy foods containing OBRS also had higher As concentrations than equivalent products that did not contain OBRS. As_i_ was the main As species in most food products tested in this study.

Conclusions: There are currently no U.S. regulations applicable to As in food, but our findings suggest that the OBRS products we evaluated may introduce significant concentrations of As_i_ into an individual’s diet. Thus, we conclude that there is an urgent need for regulatory limits on As in food.

Arsenic (As) is an established carcinogen based on studies of populations consuming contaminated drinking water (Smith et al. 2002). Recently, attention has focused on As exposure from food, in particular fruit juices (Rock 2012) and rice (Stone 2008). Rice may contain As in total concentrations up to 100–400 ng/g, including both inorganic As (As_i_) and the organic species dimethyl-arsenate (DMA) (Williams et al. 2005), with much lower concentrations (relative to DMA) of mono-methyl-arsenate (MMA) also occasionally detected. Total As (As_total_) in rice and relative proportions of DMA and As_i_ differ both geographically (Meharg et al. 2009) and as a function of genetic and environmental controls (Norton et al. 2009).

As-_i_ is more toxic than DMA or MMA (Le et al. 2000), and food regulatory limits, where they exist, are based on As_i_. Infants fed rice cereal at least once daily may exceed the daily As exposure limit of 0.17 µg/kg body weight per day based on drinking water standards (Meharg et al. 2008b). Rice products such as cereals and crackers (Sun et al. 2009) and rice drinks (Meharg et al. 2008a) are potentially significant dietary sources of As. Infants and young children are especially vulnerable because their dietary As exposure per kilogram of body weight is 2–3 times higher than that of adults [European Food Safety Authority (EFSA) 2009].

DMA is a metabolite of As_i_. Although considered less toxic than As_i_, its toxicological potential has not been studied extensively. The presence of DMA in rice is likely due to natural soil microbial processes; however, DMA was used as a pesticide before being banned by the U.S. Environmental Protection Agency (EPA) in 2009 (U.S. EPA 2009). Organic food consumers may therefore object to its presence in organic foods even in the absence of direct evidence of human health effects of DMA.

In the United States, organic brown rice syrup (OBRS) is used as a sweetener as a healthier alternative to high-fructose corn syrup in products aimed at the “organic foods” market. Added sugar is often the main ingredient in infant and toddler formula, and the addition of sucrose to a main-brand organic formula was the feature of a popular press article in relation to possible childhood obesity (Moskin 2008). Many products—including some baby milk formulas, cereal bars, and high-energy performance products for athletes—list OBRS as the major ingredient. Brown rice is usually higher in both As_total_ and As_i_ than white rice because As_i_ is localized to the aleurone layer, which is removed when rice is polished, whereas DMA passes into the grain (Carey et al. 2011; Sun et al. 2008). Ranges of As concentration in rice products, including OBRS, are similar to As concentrations in brown rice (Signes-Pastor et al. 2009).

We posit that consumers of organic food products are generally attempting to make educated eating choices and that this consumer group would be particularly interested to know if, and to what extent, OBRS introduces As_i_, DMA, and MMA into these products. We therefore measured As_total_ and As speciation in three commercially available brown rice syrups, 15 infant formulas without OBRS, 2 toddler formulas with OBRS, 29 cereal bars (13 with OBRS), and three flavors of a high-energy performance product.

## Materials and Methods

We purchased three commercial OBRSs from local or online stores. For one syrup, two bottles of the same product (from different lots) were tested. Fifteen infant formulas and two toddler formulas (initially purchased as part of a parallel study on As content of formulas and infant foods), as well as 29 cereal bars and three energy shot blocks were all purchased from local stores in the Hanover, New Hampshire, area.

*Sample preparation.* All samples were analyzed for As_total_, and selected samples were extracted for As species. For formulas, As_total_ was determined after closed vessel micro-wave digestion (MARSXpress; CEM Corp., Matthews, NC) with Optima HNO_3_. Approximately 0.25 g formula was digested in 2 mL 50% HNO_3_ (nitric acid). The samples were heated at 180°C for 10 min, allowed to cool, and then diluted to 10–25 mL with deionized water. Cereal bars and energy blocks were homogenized using a ceramic knife and were not dried before digestion. A sub-sample was digested in 2–3 mL Optima HNO_3_ and heated at 95°C for 30 min. The digested sample was diluted with deionized water to 25–50 mL. This digested sample was diluted a further 10× before analysis to reduce the acid concentration in the sample to < 5%. All digestions and dilutions were recorded gravimetrically. Samples were extracted for As speciation using 1% HNO_3_ and open-vessel heating in a micro-wave digestion unit following a heating profile of 55°C for 5 min, 75°C for 5 min, and 95°C for 20 min (Foster et al. 2007; Huang et al. 2010). An aliquot of the extracted sample was then centrifuged at 13,300 rpm for 30 min; an aliquot of that supernatant was further spin filtered at 10 kDa.

*As_total_ and As speciation.* As_total_ was determined by inductively coupled plasma mass spectrometry (ICP-MS; model 7700x; Agilent, Santa Clara, CA) using helium as a collision gas at a flow rate of 4.5 mL/min. Samples were analyzed by either external calibration or the method of standard additions. As speciation of the 1% HNO_3_ extracts was determined by ion chromatography coupled to ICP-MS using a Hamilton PRP X100 anion exchange column (Hamilton Company, Reno, NV) and a 20 mM ammonium phosphate eluant at pH 8. Formulas were evaluated in triplicate, and 5% duplicate and duplicate spikes were performed for the cereal bars and energy blocks.

We used NIST Standard Reference Material (SRM) 1568a rice flour (National Institute of Standards and Technology, Gaithersburg, MD) as a quality control material for both As_total_ measurements and As speciation. Although As species are not certified for SRM 1568a, reproducible consensus values have been demon-strated in many studies (Meharg and Raab 2009; Raab et al. 2009; Williams et al. 2005). We determined As_total_ in SRM 1568a to be 279 ± 31 ng/g (mean ± 1 SD; *n* = 6); the certified value is 290 ± 30 ng/g. For As speciation (*n* = 5), we determined DMA to be 186 ± 21 ng/g, MMA to be 9.4 ± 3.7 ng/g, and As_i_ to be 101 ± 15 ng/g, which are in the range reported by other studies.

*Data analyses.* Given our calculated values for As speciation in the formulas, we estimated As concentrations (micrograms per liter) of reconstituted formulas assuming that one scoop of powdered formula weighs 8.75 g and that one scoop of formula is added to 60 mL As-free water to make 2 fluid ounces of formula. We then estimated daily intake of As species for a baby weighing 6 kg and 9 kg, assuming consumption of six 4-ounce bottles of milk formula each day, and compared this with “safe” levels estimated for consumption of drinking water containing As_i_ at the U.S. EPA and World Health Organization (WHO) maximum contaminant limit of 10 µg/L (Meharg et al. 2008b).

## Results and Discussion

*Rice syrups.* As_total_ concentrations in three rice syrups (and from two lots of one of the syrups) ranged from 80 to 400 ng/g (Table 1). As_i_ was 80–90% of As_total_ for two of the three syrups; for the third syrup, only 50% of As_total_ was As_i_. However, because this syrup was much higher in As_total_, it also had the highest As_i_ concentration of the syrups. All syrups had detectable MMA, ranging from 3 to 4% of As_total_, but the major organic As species for each syrup was DMA. Our results are similar to those of Signes-Pastor et al. (2009) who reported dry weight As_total_ concentrations of 80, 100, 120, and 330 ng/g in four rice syrups, with 71% As_i_ and 85% extraction efficiency in the highest As syrup. Moreover, given these authors’ estimate of 15% moisture content for the syrups, we estimate that the actual contribution to As concentration in food products that include OBRS as the dried product—such as toddler formulas—would be approximately 1.15 times the concentration listed in Table 1.

**Table 1:** As concentrations and As speciation for three OBRSs.

Astotal [ng/g (mean ± 1 SD)]	Speciation analysis						
	Sample	Asi (%)	DMA (%)	MMA (%)	Sum of As species (ng/g)
A, lot 1		78 ± 6		89		7		4		81
A, lot 2		94 ± 8		84		12		4		94
B		136 ± 3		91		6		3		118
C		406 ± 6		51		46		3		294
Analyses were performed in triplicate.										

**Figure 1:**
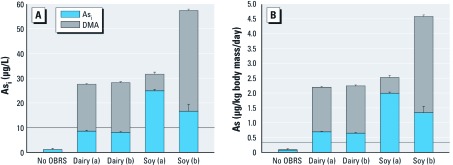
As_i_ and DMA concentrations in milk formulas with and without OBRS. (*A*) Concentrations of As_i_ and DMA in prepared formula in reconstituted milk formulas relative to the current WHO and U.S. EPA drinking water standard of 10 µg/L (horizontal line). (*B*) Daily As intake for a 9‑kg baby drinking six 4‑ounce bottles of milk formula reconstituted with As-free water relative to a 60‑kg adult drinking 2 L tap water at the safe drinking water limit (horizontal line). Data are mean ± SD. The No OBRS bars are calculated from 15 different main-brand milk formulas (Jackson et al. 2012); the OBRS bars are based on triplicate analysis from one lot (a or b) of each type.

*Baby formulas.* We analyzed 17 different formulas. Average As_total_ concentrations in the 15 infant formulas that did not contain OBRS were relatively low, in the range of 2–12 ng/g (Jackson et al. 2012). Those results were consistent with two other studies of As in infant formula (Ljung et al. 2011; Vela and Heitkemper 2004). However, the As concentrations in the two toddler formulas that listed OBRS as the primary ingredient (one dairy-based and one soy-based) were > 20 times the As concentrations in infant formulas that did not contain OBRS (Figure 1A). The proportion of As_i_ varied among products and among lots of the soy-based formula, but the concentration of As_i_ in the reconstituted formulas with OBRS was either just below (dairy, 8–9 µg/L) or 1.5–2.5 times above (soy) the current U.S. drinking water standard (10 µg/L). In addition, the OBRS formulas contained 19–40 µg/L DMA and trace levels of MMA. Expressed as daily As intake per kilogram of body weight, the exposure of infants and toddlers drinking OBRS-containing milk products is even more apparent (Figure 1B). Using web-based search engines, we found only these two toddler formulas that used OBRS, so the number of infants using this formula is presumably a very low percentage of U.S. formula-fed infants.

Infants, in a phase of rapid development, are especially vulnerable to contaminants, and emerging data suggest that As exposure early in life may pose risks not only during childhood but also in adult life (Vahter 2009). This suggests that we need to pay particular attention to the potential for As exposure during infancy. The standards and guidelines for daily intake of As are currently a matter of debate (Meharg and Raab 2009; Meharg et al. 2008b). The WHO established a provisional maximum tolerable daily intake (PMTDI) guideline of 2.1 µg/kg/day in 1983 (Food and Agriculture Organization of the United Nations/WHO 1983). For an infant weighing either 6 or 9 kg, both of the OBRS formulas would be above this value based on As_total_; for a 6-kg infant, the soy formulas would be above the guideline based only on As_i_. It should be noted that the WHO 1983 PMTDI is based on a safe drinking water limit of 50 µg/L rather than the current limit of 10 µg/L [European Food Safety Authority (EFSA) 2009; Meharg and Raab 2009]. Currently, only China has a limit for As in food: an As_i_ limit of 150 ng/g for rice (Zhu et al. 2008). Although the OBRS toddler formulas would not exceed this limit on average, As_total_ and As_i_ concentrations of these OBRS formulas are cause for concern.

*Cereal and energy bars.* OBRS is also a popular sweetener for many cereal/energy bars and high-energy athletic performance products. Our web- and store-based market survey of 100 bars indicated that about 50% contain either OBRS (31%), other rice products (5%), or both (14%). We tested 29 bars and three types (flavors) of an energy product obtained from a local supermarket. The results for the cereal/energy bars are shown in Table 2. All of the bars had detectable As_total_ with a range of 8–128 ng/g. The 7 bars that did not list any rice product among the top five ingredients were among the 8 lowest As-containing bars we tested. The remaining bars listed at least one of four rice products (OBRS, rice flour, rice grain, and rice flakes) in the first five ingredients and had As_total_ concentrations ranging from 23 to 128 ng/g.

**Table 2:** As concentrations and speciation in 29 cereal bars, with information about their rice-based ingredients.

As content					Rice ingredients						
Sample ID	Astotal (ng/g)	Percent Asi	Percent recovery	Flakes	Grain	Flour	Syrup
29		8		—		—								
21		11		—		—								
14		12		—		—								
28		12		—		—								
22		22		—		—								
8		23		—		—						ü (4)		
4		27		—		—								
17		27		—		—								
2		28		—		—						ü (4)		ü (1)
20		30		—		—								ü (3)
1		33		—		—						ü (3)		
5		34		—		—								ü (5)
9		35		—		—						ü (4)		ü (2)
101		41		—		—						ü (2)		ü (1)
7		45		92		77								ü (2)
18		51		53		119				ü (4)				
19		55		38		120						ü (1)		ü (2)
26		56		73		101						ü (4)		ü (2)
103		57		—		—						ü (2)		ü (1)
13		61		76		77								ü (2)
12		64		73		76								ü (3)
16		66		81		97				ü (1)				ü (2)
11		76		75		67				ü (4)		ü (5)		
10		83		81		71						ü (2)		ü (1)
102		86		—		—						ü (2)		ü (1)
15		90		85		124				ü (1)				ü (2)
27		101		79		97						ü (3)		ü (1)
3		119		57		85						ü (1)		ü (2)
6		128		62		113		ü (1)				ü (4)		ü (2)
—, sample was not speciated. Check marks indicate the presence of a rice-based ingredient (flakes, grain, flour, or brown rice syrup), and numbers in parentheses indicate the order of that ingredient in the ingredients list (only the first five listed ingredients were considered).														

We analyzed As speciation in 12 of the rice-containing bars. Of the 12 bars, 11 contained As_i_ concentrations > 50%, with an average of 70% As_i_. All organic As was DMA. The percent recovery (sum of As species as a percentage of As_total_) ranged from 67% to 124%; however, some of this variability is because the bars were not dried before analysis and were analyzed “as is,” with limited homogenization using a ceramic-bladed knife. The amount of As_i_ ingested when eating one of these bars is a function of the As concentration of the bar and the size (weight) of the bar. The bars we analyzed ranged in weight from 28 to 68 g; at the upper limit of bar weight and As_i_ content, an individual bar contained up to 4 µg As_i_. For example, bar 27 weighed 45 g and contained 101 ng/g As_total_ and 79% As_i_, equating to an As_i_ content of 3.6 µg.

*Energy shot blocks.* We also analyzed As concentration and speciation in three high-energy products for endurance athletes known as “energy shot blocks," each of which contained OBRS. Although an educated consumer might be aware of the potential for rice to contain As (and therefore know that products containing rice ingredients might also contain As), the energy shot blocks are gel-like blocks, so it would not be immediately apparent to the consumer that these too are rice-based products.

The As concentration in one of the energy shot blocks containing OBRS was 84 ± 3 ng/g As_total_ (*n* = 3), which was 100% As_i_. The other two energy shot blocks were very similar to one another in As_total_ concentrations (171 ± 3.6 ng/g, mean ± SD; *n* = 6) and speciation (53% As_i_). No MMA was detected in the energy shot blocks. All three flavors contained 2.5–2.7 µg As_i_ per 30-g serving. The manufacturer recommends consuming up to two servings (60 g) per hour during exercise, so an endurance athlete consuming four servings during a 2-hr workout would consume approximately 10 µg As_i_ per day, equal to the As_i_ intake resulting from consumption of 1 L of water at the current U.S. EPA and WHO limit of 10 µg/L. Athletes consuming the two flavors containing 171 ng/g As_total_ would also consume 2.5 µg DMA per 30-g serving.

## Conclusions

Food is a major pathway of exposure to As for most individuals (EFSA 2009). Rice and rice products can contribute to an individual’s As_i_ exposure (Meharg et al. 2008a, 2008b; Williams et al. 2005). There is a growing body of information about As concentration and speciation in rice in the peer-reviewed literature and thus in the public domain, but much less information is available on rice-based food products. Rice products are used in a variety of foods, including gluten-free products and, as we show here, in products where OBRS is used as an alternative to high-fructose corn syrup. The formulas containing OBRS—which could be the sole sustenance for an individual over a critical period of develop-ment—can result in consumption of milk with As concentrations much higher than the drinking water standard, yet there are no U.S. regulations to deal with this particular scenario. Similarly, endurance athletes who consume 4 servings of OBRS-containing energy shot blocks (manufacturer-recommended maximum for 2 hr of physical activity) may be exposed to as much as 10 µg As_i_ and 20 µg As_total_ in a single day. Moreover, the major As species in the overwhelming majority of food products we have tested is the more toxic As_i_, a finding that, although noted in other studies (Sun et al. 2009), is particularly troubling given the non-threshold relation-ships between cancer risk and exposure to As_i_ (National Research Council 2001).

There are currently no U.S. regulations applicable to As in food, but our findings suggest that the OBRS-containing products we evaluated may introduce significant concentrations of As_i_ into an individual’s diet. Thus, we conclude that there is an urgent need for regulatory limits on As in food.
